# Urinary Tamm‐Horsfall protein, albumin, vitamin D‐binding protein, and retinol‐binding protein as early biomarkers of chronic kidney disease in dogs

**DOI:** 10.14814/phy2.13262

**Published:** 2017-06-02

**Authors:** Fernanda Chacar, Márcia Kogika, Talita R. Sanches, Douglas Caragelasco, Cínthia Martorelli, Camila Rodrigues, Jose Manuel C. Capcha, Dennis Chew, Lúcia Andrade

**Affiliations:** ^1^ Department of Veterinary Internal MedicineSchool of Veterinary Medicine and Animal ScienceUniversity of São PauloSão PauloBrazil; ^2^ Division of NephrologySchool of MedicineUniversity of São PauloSão PauloBrazil; ^3^ Department of Veterinary Clinical SciencesOhio State UniversityColumbusOhio

**Keywords:** Chronic kidney disease, dogs, retinol‐binding protein, Tamm‐Horsfall protein, vitamin D‐binding protein

## Abstract

Proteinuria is a marker and mediator of chronic kidney disease (CKD). In clinical practice, the urinary protein‐to‐creatinine ratio (UP/C) is of limited usefulness, because it indicates only the magnitude of proteinuria and not the origin of the loss (glomerular or tubular). The complete assessment of proteinuria includes quantitative and qualitative evaluations, both of which are required in order to optimize the therapy. In addition to measuring the UP/C, we performed SDS‐PAGE and western blotting to determine the expression of albumin, vitamin D‐binding protein (VDBP), retinol‐binding protein (RBP), and Tamm‐Horsfall protein (THP) in urine samples of 49 dogs: healthy (control) dogs (*n *=* *9); and dogs with CKD (*n *=* *40), stratified by stage. In the dogs with stage 3 or 4 CKD, there was a predominance of tubular proteins. Neither VDBP nor RBP was observed in the urine of the control dogs. Among the dogs with stage 1 or 2 CKD, VDBP and RBP were detected in those without proteinuria or with borderline proteinuria. The expression of urinary albumin was significantly higher in the stage 4 group than in any other group (*P *≤* *0.01). In the stage 4 group, urinary THP was either undetectable or lower than in the control group (*P *≤* *0.01). In conclusion, urinary VDBP and RBP might act as early markers of kidney injury, and a decrease in urinary THP could be an indicator of CKD progression.

## Introduction

The optimal evaluation of proteinuria includes quantitative and qualitative assays. The urinary protein‐to‐creatinine ratio (UP/C) provides information only about the magnitude of proteinuria, not about its origin. The nature of the proteinuria can indicate the origin (glomerular, tubular, or both) and severity of nephron injury, and the identification of the injured nephron segment is essential to ensuring adequate treatment (Yalçin and Çetin 2004).

The process of identifying and determining the severity of chronic kidney disease (CKD) typically focuses on the glomerulus. The severity of CKD is characterized by determining the serum creatinine level, which allows the glomerular filtration rate to be estimated, and the degree of albuminuria, which is generally accepted as a measure of glomerular permeability. These biomarkers reflect the filtration and barrier functions of the kidney glomeruli, although small albuminuria can also result from tubule dysfunction (Russo et al. 2007). Given that the proximal tubules make up approximately 90% of the renal cortical mass and that proximal tubule injury can lead to fibrosis, glomerulosclerosis, and CKD (Grgic et al. 2002), we evaluated whether sensitive, specific markers of proximal tubule injury—vitamin D‐binding protein (VDBP) and retinol‐binding protein (RBP)—can identify CKD in dogs. Recent genome‐wide association studies have identified downregulated Tamm‐Horsfall protein (THP) expression as a risk factor for CKD and have suggested that the urinary expression of THP could be useful as a biomarker of CKD progression (Köttgen et al. 2010).

Microalbuminuria has been described as an early marker of kidney disease, and macroalbuminuria has been described as a marker of the glomerular injury that has been associated with the progression of CKD (Grauer 2005; Birn and Christensen 2006; Bacic et al. 2010). VDBP is the main carrier of vitamin D and its metabolites in the circulation. After being freely filtered through the glomerulus, the VDBP/25‐OH‐vitamin D complex is taken up through megalin/cubilin‐mediated endocytosis on the brush border of the proximal tubules. The VDBP complex is degraded inside the tubular cell, and calcidiol binds to an intracellular VDBP, which interacts with 1‐*α*‐hydroxylase or with 24‐hydroxylase resulting in 1,25(OH)2‐vitamin D (calcitriol; active form of vitamin D) or 24,25(OH)2‐vitamin D (an inactive form of vitamin D), respectively (de Brito Galvao et al. 2013). Because the proximal tubules are the only site for reabsorption of the complex, the presence of VDBP in urine can indicate proximal tubular dysfunction and can be a marker of interstitial tubular fibrosis (Nykjaer et al. 2001; Mirković et al. 2013). Some studies have suggested that the massive loss of VDBP in urine, as has been observed in humans with nephrotic syndrome, can lead to vitamin D deficiency. Although that hypothesis remains controversial, vitamin D deficiency has been reported to be associated with CKD progression (González et al. 2004; Ravani et al. 2009; Pilz et al. 2011; Kendrick et al. 2012; Yousefzadeh et al. 2014).

RBP carries retinol from the liver into the peripheral circulation. After being freely filtered by the glomerulus, it is reabsorbed in the proximal tubules by megalin/cubilin‐mediated endocytosis. Proximal tubular dysfunction can lead to the loss of RBP in urine, such loss being associated with proximal tubular injury and tubulointerstitial fibrosis. The urinary RBP expression is also correlated with the extent of tubular interstitial fibrosis in dogs and humans (Forterre et al. 2004; Pallet et al. 2014).

Under physiological conditions, THP is synthesized exclusively by the epithelial cells of the thick limb of Henle's loop and by the distal convoluted tubules. Therefore, the presence of THP is an expected finding in the urine of healthy individuals. The loss of functional renal mass, together with tubular dysfunction, can result in a dramatic reduction in, or even the absence of THP expression in urine, and a decrease in urinary expression of THP can be seen as renal disease progresses (Raila et al. 2014).

The hypotheses of this study were that quantitative and qualitative assays are both necessary for the optimal evaluation of proteinuria, and that identification of specific urinary proteins might provide better information about the site of the injured nephron. Therefore, the aim was to evaluate albumin, VDBP, RBP, and THP in the urine of dogs with CKD, stratified by stage.

## Materials and Methods

### Study design

This study was approved by the Animal Research Ethics Committee of the University of São Paulo School of Veterinary Medicine and Animal Science (Protocol no. 1383161014). Urine samples were collected with the informed consent of the dogs' owners. All of the dogs were treated at the Clinic of the Veterinary Teaching Hospital of the University of São Paulo School of Veterinary Medicine and Animal Science, in the city of São Paulo, Brazil.

We recruited nine clinically normal dogs and 40 dogs with CKD (total of 23 males and 26 females, aging 7.6 ± 0.67 years old, and of all breeds). The dogs were divided into four groups by CKD stage, according to the International Renal Interest Society (IRIS) staging criteria (IRIS 2015). The dogs in the stage 1 CKD group (*n *=* *10) were nonazotemic (serum creatinine < 1.4 mg/dL) but showed some combination of abnormal renal ultrasound findings (kidneys of decreased size, with regular or irregular contours; reduced demarcation between the renal cortex and medulla; and diffuse hyperechogenicity), isosthenuria, or proteinuria (UP/C > 0.5). The stage 2 CKD group (*n *=* *10) comprised dogs with mild renal azotemia (serum creatinine, 1.4–2.0 mg/dL) and ultrasound findings indicative of chronic nephropathy, isosthenuria, or proteinuria. The stage 3 CKD group (*n *=* *10) comprised dogs with moderate renal azotemia (serum creatinine, 2.1–5.0 mg/dL), together with mild to moderate clinical signs as polyuria and polydipsia. The stage 4 CKD group (*n *=* *10) comprised dogs with severe renal azotemia (serum creatinine > 5.0 mg/dL) and showing clinical signs similar to those of the dogs in the stage 3 CKD group, together with weight loss, decreased appetite, and gastrointestinal disturbance. Serum samples for creatinine measurements were collected from all of the dogs. A control group (*n *=* *9) comprised clinically healthy dogs with normal findings from history and physical examination as well as in the complete blood count (CBC), normal serum biochemical profiles (urea, creatinine, alanine aminotransferase, alkaline phosphatase, aspartate aminotransferase, albumin, total protein, total calcium, phosphate, sodium, potassium, and chloride), and normal abdominal ultrasound findings, including those related to the kidneys.

Dogs were classified as nonproteinuric (UP/C < 0.2), borderline proteinuric (0.2 ≤  UP/C ≤ 0.5), or proteinuric (UP/C > 0.5) according to the 2015 IRIS guidelines (IRIS 2015). We excluded those with any of the following proteinuria‐associated diseases: systemic hypertension, pyoderma, neoplasia, Cushing syndrome, diabetes mellitus, urinary tract infection, urolithiasis, prostatitis, pyometra, and parasitemia (ehrlichiosis and babesiosis).

To confirm the diagnosis of CKD and to stage the disease, the dogs were submitted to a physical examination, arterial blood pressure measurement, blood sampling, urine sampling, and abdominal (renal) ultrasound. Dogs in the control group underwent the same evaluations in order to confirm their healthy state. Blood pressure was measured by Doppler method, in accordance with the guidelines American College of Veterinary Internal Medicine (Brown et al. 2007).

### Specimen collection and processing

Blood samples were collected for serum urea and creatinine measurements with commercial kits (FS 1 3101; DiaSys Diagnostic Systems, Holzheim, Germany, for urea, and K 96; Labtest Diagnóstica, Lagoa Santa, Brazil, for creatinine). All measurements were made in an automated biochemical analyzer (Labmax 240; Labtest Diagnóstica). Urine samples were obtained by ultrasound‐guided cystocentesis or by aseptic urethral catheterization, and the urine collected was processed immediately for urinalysis and culture. In brief, the urine was centrifuged (168 × ***g*** for 10 min), after which the supernatant was separated into 1‐ml aliquots and stored at −80°C for no more than 1 year. Those aliquots were used in order to measure creatinine and total protein concentrations, as well as to perform SDS‐PAGE and Western blotting. Urinary protein concentrations were measured by the Bradford method (Bradford 1976), and urinary creatinine concentration was determined by the modified Jaffe technique (Lustgarden and Wenk 1972). The UP/C was then calculated.

### SDS‐PAGE

In order to identify the electrophoretic bands of urinary proteins, we used a constant current of 40 mA in stacking (4%) and separation gels (10%). The protein bands were stained with silver nitrate. We used a low‐range SDS‐PAGE molecular‐weight standard (161‐0375, Precision Plus Protein Kaleidoscope; Bio‐Rad, Hercules, CA). Based on the electrophoretic patterns and using the molecular weight of albumin (approximately 60 kDa) as a cut‐off point, we classified proteins as low molecular weight (<60 kDa) or high molecular weight (>60 kDa). High‐molecular‐weight proteins were considered to be of glomerular origin (hereafter referred to as glomerular proteins), whereas low**‐**molecular‐weight proteins were considered to be of tubular origin (hereafter referred to as tubular proteins). The proportions of glomerular and tubular proteins were determined by densitometric analysis (VisionWorks software; Ultra‐Violet Products, Cambridge, England).

### Western blot

A variable quantity of urine, the exact volume of which was dependent on obtaining 10 *μ*g of urinary creatinine, was diluted with a buffer (3.55 mL distilled water; 1.25 mL 0.5 Trishydrochloric acid, pH 6.8; 2.5 mL glycerol; 2.0 mL 10% SDS and 0.2 mL 0.5% bromophenol blue). After 12% SDS‐PAGE, the proteins were transferred onto a polyvinylidene difluoride membrane, and the blots were blocked for 1 h with 5% skim milk diluted in Tris‐buffered saline with Tween 20 (24.2 g Tris buffer; 29.2 g NaCl; 3.36 g EDTA in 1 L distilled water). As in previous canine studies (Lustgarden and Wenk 1972; Nabity et al. 2011), the blots were then incubated overnight at 4°C with specific antibodies, diluted in Tris‐buffered saline with Tween 20: anti‐albumin (ab112986, 1:500; Abcam, Cambridge, MA), anti‐VDBP (ab95469, 1:500; Abcam), anti‐RBP (ab48624, 1:250; Abcam), and sheep anti‐human THP (AB733, 1:100; EMD Millipore Corporation, Danvers, MA). The respective horseradish peroxidase‐conjugated secondary antibodies (anti‐goat, 1:10,000; anti‐goat, 1:10,000; anti‐rabbit, 1:2000; and anti‐sheep, 1:2000) were added, and the immunoreactive bands were observed via a chemiluminescence kit (ECL;Amersham, Piscataway, NJ), in accordance with the manufacturer's instructions. The densitometric analysis of the immunoreactive bands was performed using the Image J program (National Institutes of Health, Bethesda, MD). The results, expressed as percentages, were multiplied by 100.

### Statistical analysis

Descriptive statistics were calculated for the UP/C and for the densitometry of the urinary protein bands. Depending on the results obtained on the Kolmogorov–Smirnov test, we used either one‐way ANOVA or the Kruskal–Wallis test. For the comparison of two means, Student's *t*‐test or the Mann–Whitney test was applied. For multiple comparison tests, the Tukey‐Kramer test or Dunn's test was performed. Values of *P *≤* *0.05 were considered statistically significant. The data in the graphs are presented as mean ± SEM. Statistical analysis was performed with GraphPad Prism software, version 5 (GraphPad Software Inc., San Diego, CA).

## Results

### UP/C and SDS‐PAGE

As can be seen in Table 1, the range (mean ± SEM) for UP/C in the healthy (control) dogs was 0.02–0.41 (0.14 ± 0.04). Of the nine control dogs, three showed proteinuria in the borderline range according to IRIS guidelines (UP/C 0.2 to 0.5). In dogs with CKD in stages 1, 2, 3, and 4, the mean UP/C was, respectively, 0.02–3.01 (0.39 ± 0.29), 0.01–1.14 (0.33 ± 0.12), 0.07–4.57 (1.51 ± 0.54), and 1.40–6.94 (4.37 ± 0.47). Of the ten dogs in the stage 4 group, nine had a UP/C above 2.0. In terms of the UP/C, there were significant differences among the various groups (*P *≤* *0.0001), between the control group and the stage 4 group (*P *≤* *0.001), between the stage 1 and stage 4 groups (*P *≤* *0.001), and between the stage 2 and stage 4 groups (*P *≤* *0.01). The mean UP/C for the control group did not differ significantly from that obtained for the stage 1, stage 2, or stage 3 group.

**Table 1:** Degree of proteinuria, as quantified by the urinary protein‐to‐creatinine ratio, in clinically healthy (control) dogs and in dogs with chronic kidney disease, stratified by stage

	Healthy dogs	Dogs with CKD			
		Stage 1	Stage 2	Stage 3	Stage 4
	UP/C	UP/C	UP/C	UP/C	UP/C
Mean	0.14	0.39	0.33	1.51	4.37
SD	0.14	0.92	0.38	1.72	1.50
SEM	0.04	0.29	0.12	0.54	0.47
Median	0.04	0.05	0.14	0.63	4.25
Minimum	0.02	0.02	0.01	0.07	1.40
Maximum	0.41	3.01	1.14	4.57	6.94

CKD, Chronic kidney disease; UP/C, Urinary protein‐to‐creatinine (ratio).

**Figure 1:**
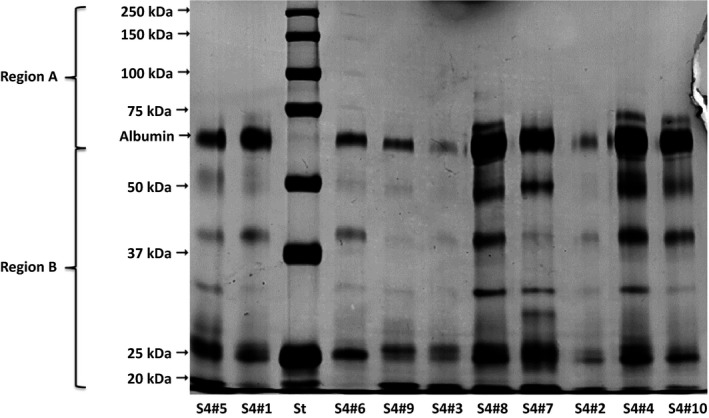
SDS‐PAGE of urinary proteins in ten dogs (by #) with stage 4 chronic kidney disease (S4).

In the control group, the urinary proteins were predominantly tubular, seven of the ten control group dogs showing a higher proportion of urinary protein bands in the < 60 kDa (tubular) range than in the > 60 kDa (glomerular) range (Table 2): the glomerular proteins had 1–2 protein bands, and the tubular proteins had 1–7 bands. In the stage 1 group, there was a predominance of tubular proteins, ≥3 tubular protein bands (20.1–58.1 kDa) being observed in eight of the ten dogs in the group. As can be seen in Table 2, the proportion of urinary protein bands in the glomerular range was significantly lower than was that of those in the tubular range (*P *=* *0.0274). In the stage 2 group, the glomerular and tubular patterns were detected equally, not only in terms of the proportional distribution of the glomerular and tubular proteins but also in terms of the molecular weights of the proteins (Table 2). In five of the ten dogs in that group, glomerular proteins accounted for more than 50% of the urinary proteins; in the dogs in which there were 1–2 glomerular protein bands, the bands ranged from 71.9 kDa to 128.1 kDa. Tubular proteins accounted for more than 50% of the urinary proteins in the remaining five dogs in the stage 2 group; in three of those five dogs, there were ≥ 4 low‐molecular‐weight protein bands (range, 24.8–59.0 kDa). There was no significant difference between the proportions of glomerular and tubular proteins in the stage 2 group (*P *=* *0.54). In the stage 3 group, there was a predominance of tubular proteins, which accounted for more than 50% (range, 52.4–84.0%) of the urinary proteins in all ten of the dogs in the group (Table 2), and the overall proportion of tubular proteins was significantly higher than was that of glomerular proteins (*P *≤* *0.0001). In each of the stage 3 group dogs, there were 4–7 low‐molecular‐weight protein bands (range, 21–59 kDa). In the stage 4 group, tubular proteins also predominated (Fig. 1), the proportion of tubular proteins ranging from 62.6% to 79.6% (mean ± SEM, 71.2 ± 1.8%), and there were 3–6 low‐molecular‐weight protein bands (57.5–20.1 kDa). Among the dogs with stage 4 CKD, there was a significant difference between the proportion of tubular proteins and that of glomerular proteins (*P *≤* *0.0001).

**Table 2:** Proportional distribution of urinary proteins of glomerular origin (MW > 60 kDa) and of tubular origin (MW < 60 kDa), based on SDS‐PAGE analysis, in clinically healthy (control) dogs and in dogs with chronic kidney disease, stratified by stage

	Proteins of glomerular origin (>60 kDa)					Proteins of tubular origin (<60 kDa)				
	Healthy dogs	Dogs with CKD				Healthy dogs	Dogs with CKD			
		Stage					Stage			
		1	2	3	4		1	2	3	4
	(%)	(%)	(%)	(%)	(%)	(%)	(%)	(%)	(%)	(%)
Mean	34.7	43.5	48.0	30.3	28.8	65.3	56.5	52.0	69.7	71.2
SD	11.4	12.0	14.2	8.9	5.6	11.4	12.0	14.2	8.9	5.6
SEM	3.8	3.8	4.4	2.8	1.7	38.0	3.7	4.4	2.8	1.7
Median	32.5	30.9	26.2	16.0	20.3	67.5	33.6	28.6	52.4	62.6
Minimum	17.4	41.3	50.2	30.9	27.6	44.2	58.7	49.7	68.8	72.3
Maximum	55.8	66.3	71.3	47.6	37.8	82.6	69.1	73.8	84.0	79.6

CKD, Chronic kidney disease.

### Serum creatinine levels

The serum creatine level (mean ± SEM) was 0.93 ± 0.04 mg/dL in the control group, 1.01 ± 0.06 mg/dL in the stage 1 group, 1.75 ± 0.05 mg/dL in the stage 2 group, 3.14 ± 0.25 mg/dL in the stage 3 group, and 7.95 ± 0.85 mg/dL in the stage 4 group.

### Western Blot

#### Albumin

**Figure 2:**
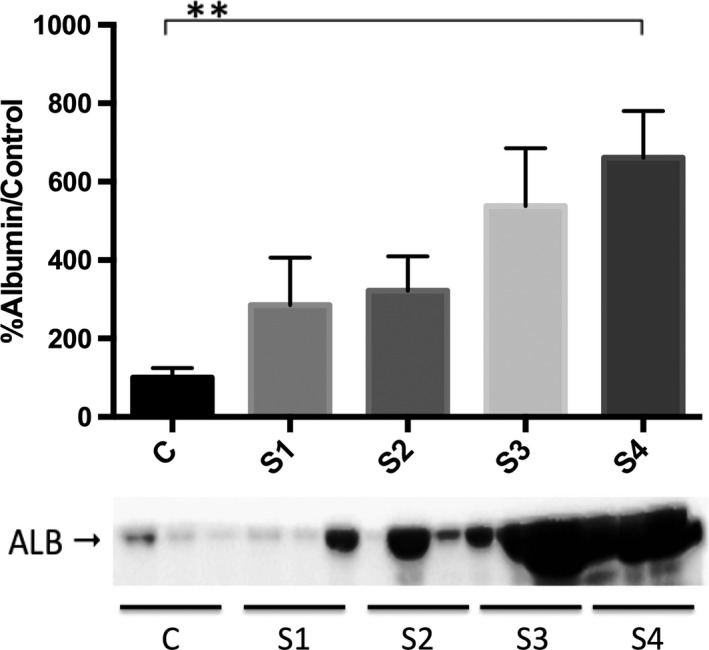
Densitometric analysis of urinary albumin by Western blot in clinically healthy (control) dogs and in dogs with chronic kidney disease (CKD), according to the International Renal Interest Society staging criteria. C: control; S1: stage 1 (CKD); S2: stage 2 (CKD); S3: stage 3 (CKD); S4: stage 4 (CKD). **P ≤* *0.01 versus all other groups.

The densitometric analysis showed that the range (mean ± SEM) for urinary albumin was 28.2–243.4% (100.0 ± 24.9%) in the control group, 30.0–1279.0% (285.2 ± 121.3%) in the stage 1 group, 37.3–910.4% (321.8 ± 87.5%) in the stage 2 group, 33.2–1303.0% (538.1 ± 147.3%) in the stage 3 group, and 301.9–1303.0% (661.4 ± 119.1%) in the stage 4 group. As can be seen in Figure 2, the difference among the groups, in terms of urinary albumin expression, was significant (*P *=* *0.009).

#### VDBP

**Figure 3:**
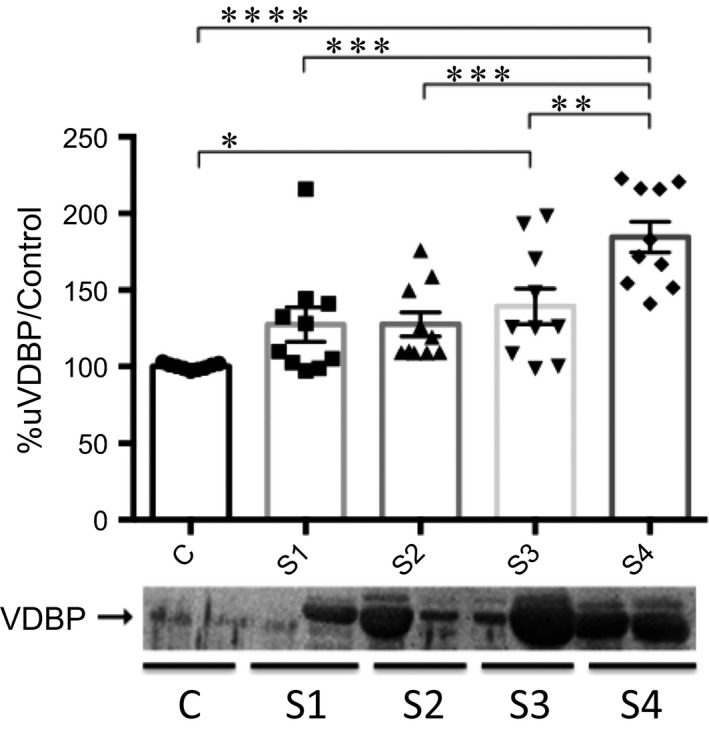
Densitometric analysis of urinary vitamin D‐binding protein (VDBP) by Western blot in clinically healthy (control) dogs and in dogs with chronic kidney disease (CKD), according to the International Renal Interest Society staging criteria. C: control; S1: stage 1 (CKD); S2: stage 2 (CKD); S3: stage 3 (CKD); S4: stage 4 (CKD). *P ≤* *0.05; **P ≤* *0.01; ***P ≤* *0.001; ****P ≤* *0.0001.

The range of values (mean ± SEM) obtained in the densitometric analysis for urinary VDBP was 97.1–103.0% (100 ± 0.64%) in the control group, 97.2–216.0% (127.6 ± 11.3%) in the stage 1 group, 109–175.9% (127.7 ± 7.84%) in the stage 2 group, 98.7–198.2% (139.4 ± 11.58%) in the stage 3 group, and 141.2–222.8% (184.5 ± 10.1%) in the stage 4 group. The ANOVA detected a significant difference among the groups (*P *≤* *0.0001). The Tukey‐Kramer test revealed significant differences between the control and stage 3 groups (*P *≤* *0.05), between the control and stage 4 groups (*P *≤* *0.0001), between the stage 1 and stage 4 groups (*P *≤* *0.001), between the stage 2 and stage 4 groups (*P *≤* *0.001), and between the stage 3 and stage 4 groups (*P *≤* *0.01). The densitometric analysis of the immunoreactive bands showed that urinary VDBP was present in urine even in the early stages of CKD (Fig. 3). Among the ten dogs with stage 1 CKD, VDBP was detected in the urine of seven and the UP/C was normal (range, 0.04–0.38) in six, proteinuria (UP/C, 3.01) being detected in only one. Among the ten dogs with stage 2 CKD, urinary VDBP was detected in nine and the UP/C was normal (range, 0.02–0.48) in seven, proteinuria being detected in the two remaining dogs (UP/C, 1.14 and 0.72, respectively).

#### RBP

**Figure 4:**
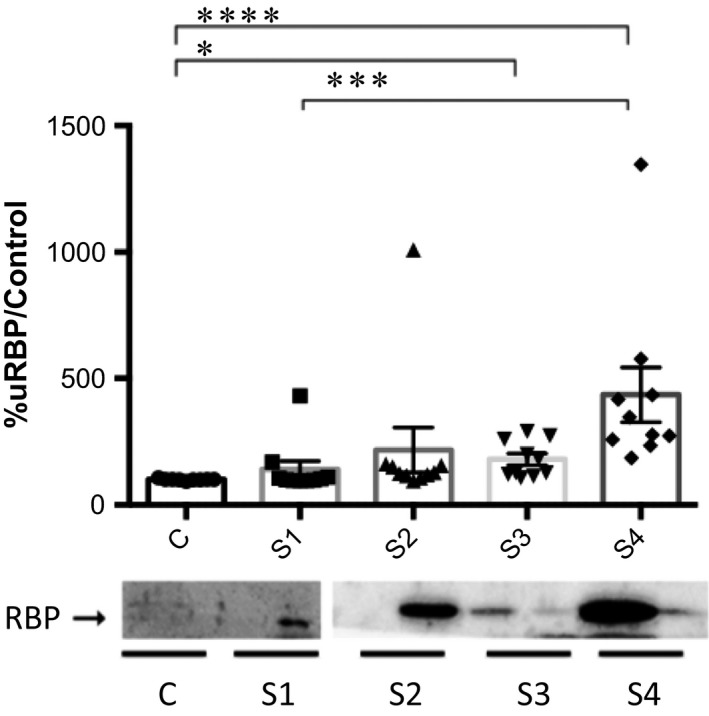
Densitometric analysis of urinary retinol‐binding protein (RBP) by Western blot in clinically healthy (control) dogs and in dogs with chronic kidney disease (CKD), according to the International Renal Interest Society staging criteria. C: control; S1: stage 1 (CKD); S2: stage 2 (CKD); S3: stage 3 (CKD); S4: stage 4 (CKD). *P ≤* *0.05; ***P ≤* *0.001; ****P ≤* *0.0001.

The densitometric analysis showed that the range (mean ± SEM) for urinary RBP was 93.7–106.3% (100.0 ± 1.2%) in the control group, 92.4–431.0% (139.4 ± 33.0%) in the stage 1 group, 95.7–1010.0% (215.3 ± 88.5%) in the stage 2 group, 106.9–291.2% (177.4 ± 21.7%) in the stage 3 group, and 185.9–1347.0% (432.4 ± 108.2%) in the stage 4 group. The Kruskal–Wallis test revealed a significant difference among the groups (*P *≤* *0.0001), and Dunn's test showed differences between the individual groups: control versus stage 3 (*P *≤* *0.05); control versus stage 4 (*P *≤* *0.0001); and stage 1 versus stage 4 (*P *≤* *0.001). However, as shown in Figure 4, the densitometric analysis of the immunoreactive bands showed that urinary RBP was present in two of the ten dogs with stage 1 CKD, one of which was proteinuric (UP/C, 3.01).

#### THP

**Figure 5:**
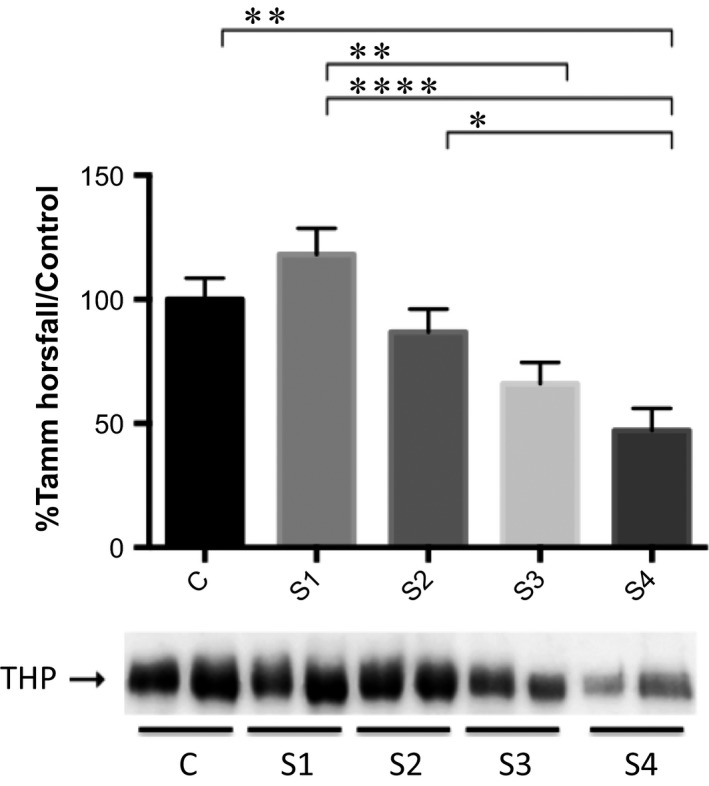
Densitometric analysis of Tamm‐Horsfall protein by Western blot in clinically healthy (control) dogs and in dogs with chronic kidney disease (CKD) according to the International Renal Interest Society staging criteria. C: control; S1: stage 1 (CKD); S2: stage 2 (CKD); S3: stage 3 (CKD); S4: stage 4 (CKD). *P ≤* *0.05; **P ≤* *0.01; ****P ≤* *0.0001.

The range of values (mean ± SEM) obtained in the densitometric analysis for urinary THP was 56.3–135.3% (100.0 ± 7.7%) in the control group, 70.4–185.1% (117.2 ± 10.8%) in the stage 1 group, 43.1–136.4% (86.0 ± 9.0%) in the stage 2 group, 33.8–131.4% (65.4 ± 8.7%) in the stage 3 group, and 12.0–100.6% (46.7 ± 9.0%) in the stage 4 group. The ANOVA detected a significant difference among the groups (*P *≤* *0.0001). The Tukey–Kramer test revealed significant differences between the control and stage 4 groups (*P *≤* *0.01), between the stage 1 and stage 3 groups (*P *≤* *0.01), between the stage 1 and stage 4 groups (*P *≤* *0.0001), and between the stage 2 and stage 4 groups (*P *≤* *0.05). The urinary expression of THP was found to decrease in parallel with the progression of CKD (Fig. 5).

## Discussion

According to the IRIS guidelines, borderline proteinuria is defined as a UP/C within the 0.2–0.5 range (IRIS 2015). Therefore, some (3/9) of the control group dogs in this study could be classified as having borderline proteinuria, although no urinary VDBP was detected in any of those dogs. In a previous electrophoretic study (Yalçin and Çetin 2004), healthy dogs typically show a predominance of tubular proteins, similar to what was detected in our study. Control dogs of our study showed minimal albumin expression as detected by Western blotting. This is an expected finding, given that albumin is known to account for 40–60% of the total proteins found in urine of dogs (Yalçin and Çetin 2004).

Urinary VDBP and RBP levels have been reported in cubilin‐deficient dogs and in dogs with CKD, respectively, whereas there have been no reports of VDBP or RBP in the urine of healthy dogs (Nykjaer et al. 2001; Forterre et al. 2004). In the present study, urinary VDBP was detected in dogs in the early stages of CKD: in seven of the ten dogs in the stage 1 group; and in nine of the ten dogs in the stage 2 group. Of the ten dogs in the stage 1 group, seven were nonproteinuric and nonazotemic, whereas seven of the ten in the stage 2 group were nonproteinuric. These findings could support that increased urinary VDBP and RBP are early indicators of proximal tubular injury in the dog, even when UP/C and serum creatinine values are within the reference range. Use of these biomarkers could facilitate the early diagnosis of chronic renal injury and underscores the need for new markers of such injury to be introduced into routine laboratory panels.

Recent studies have shown that the determination of serum levels of symmetric dimethylarginine is more sensitive than serum creatinine in detecting a minimal decrease in the glomerular filtration rate (i.e., early renal dysfunction), and the IRIS currently recommends the measurement of the former (Braff et al. 2014; Hall et al. 2015). Because CKD tends to advance, early diagnosis of renal injury can have a direct effect on the prognosis (Levey et al. 2005).

Among the dogs with stage 2 CKD evaluated in the present study, those that were nonproteinuric or slightly azotemic (serum creatinine ≥ 1.4 and ≤ 2.0 mg/dL) presented with increased urinary VDBP and urinary RBP. These findings emphasize the role of both of those urinary proteins as markers of early chronic kidney injury. In humans with diabetes mellitus, the early development of proximal tubular injury has been evaluated by immunodetection of urinary VDBP, even before the onset of microalbuminuria, which underscores the potential importance of VDBP as an early marker of CKD/tubular injury in dogs (Tian et al. 2014).

In dogs with CKD, urinary RBP has been previously reported to be an early marker of kidney injury when it is detected in urine prior to the onset of azotemia (Forterre et al. 2004; Nabity et al. 2011). In the present study, urinary VDBP and RBP were detected in dogs with stage 1 or 2 CKD, regardless of the UP/C values. In fact, seven of the ten dogs with stage 1 CKD had a UP/C < 0.2, whereas eight of the ten dogs with stage 2 CKD had a UP/C < 0.5, those values being considered to be within the normal and borderline ranges, respectively. Therefore, our findings underscore the importance of the qualitative evaluation of urinary proteins by electrophoresis and Western blotting in allowing the detection of the specific proteins that are normally reabsorbed in the proximal convoluted tubules.

Microalbuminuria (urinary albumin excretion 30 to 300 mg/24 h) and macroalbuminuria (urinary albumin excretion > 300 mg/24 h) have been associated with the early detection of renal injury and with the progression of CKD, respectively. In this study, the quantity of urinary albumin detected by densitometric analysis of immunoreactivity was significant only in the dogs with stage 4 CKD, indicating that urinary VDBP and RBP are earlier markers of chronic kidney injury than is albuminuria. Therefore, the use of qualitative methods to fully identify urinary proteins is important and helpful for the recognition of the affected segment of the nephron, which, in turn, informs decisions regarding the appropriate management and treatment of dogs with CKD.

In this study, a decrease in urinary THP occurred mainly in dogs with stage 3 or 4 CKD and was accompanied by an increase in urinary albumin expression. Those findings are indicative of the progression of renal disease in dogs (Raila et al. 2007, 2014). In addition, we found that the intensity of urinary VDBP and RBP immunoreactivity was highest in the dogs with stage 4 CKD, which is consistent with progressive proximal tubular injury. The decrease in urinary THP expression reflects damage to the thick limb of Henle's loop and distal convoluted tubules, or even the loss of nephrons. Therefore, low urinary expression of THP might act as a marker of progressive renal tubular disease. The molecular weight of THP is 90–130 kDa, and the SDS‐PAGE showed very few bands within that range in our dogs with stage 4 CKD. Our results for urinary THP, considering both of the qualitative methods employed (electrophoresis and Western blotting), are similar to those from other studies evaluating the pathophysiology of advanced‐stage renal disease in dogs (Raila et al. 2007, 2014). Our findings support the concept that decreasing urinary THP acts as a marker of CKD progression, given that a critical loss of renal tubular mass affects the synthesis of this protein.

It has been assumed that a UP/C > 2.0 in dogs strongly reflects the expression of high‐molecular‐weight proteins, including albumin, as a consequence of a glomerular lesion (Lees et al. 2005; Brown et al. 2013). However, in the present study, the electrophoretic analysis revealed the predominance of low‐molecular‐weight proteins (i.e., proteins of tubular origin) in several dogs with proteinuria, especially in those with stage 4 CKD and a UP/C > 2.0. The finding that tubular proteins were predominant in dogs with a UP/C > 2.0 could indicate that using this cut‐off point in order to differentiate between proteins of tubular origin and those of glomerular origin is not a very reliable system. These results underscore the importance of the qualitative analysis, because the evaluation of proteinuria based solely on a quantitative method (UP/C) could lead to misinterpretations that affect the therapeutic approach (Brown et al. 2013).

It is important to note that the established cutoff< 60 kDa for tubular proteins and the cutoff> 60 kDa for glomerular proteins assumed in this study may lead to a misconception about the origin of proteinuria. Urinary proteases cleave proteins into smaller fragments embarrassing the interpretation of results. The canine urine samples were stored at −80°C for no more than 1 year, which probably inhibited the activity of proteases. However, it is not possible to assure that the low molecular weight protein bands found on SDS‐PAGE were not albumin fragments. On the other hand, western blotting immunodetected some of low molecular weight protein bands observed on SDS‐PAGE into the range of 50 kDa and 25 kDa, which corresponded to VDBP and RBP, respectively. According to these findings we could confirm that at least these protein bands were not albumin fragments. Proteomic analysis may be considered as a next step to elucidate these questionings.

Our findings suggest that qualitative and quantitative investigations are both required for the appropriate interpretation of proteinuria, and that the identification of specific proteins can facilitate the identification of the anatomical site of the nephron injury. The determination of urinary expression of VDBP and RBP can provide important information, because both of those proteins can be used as markers of early kidney injury (stage 1 or 2 CKD), whereas low urinary expression of THP can be associated with advanced‐stage renal disease (stage 3 or 4 CKD) and serve as a marker of CKD progression in dogs.

## Conflict of Interest

No conflicts of interest, financial or otherwise, are declared by the author(s).
